# Dissection of Pleiotropic QTL Regions Controlling Wheat Spike Characteristics Under Different Nitrogen Treatments Using Traditional and Conditional QTL Mapping

**DOI:** 10.3389/fpls.2019.00187

**Published:** 2019-02-26

**Authors:** Xiaoli Fan, Fa Cui, Jun Ji, Wei Zhang, Xueqiang Zhao, JiaJia Liu, Deyuan Meng, Yiping Tong, Tao Wang, Junming Li

**Affiliations:** ^1^Chengdu Institute of Biology, Chinese Academy of Sciences, Chengdu, China; ^2^Center for Agricultural Resources Research, Institute of Genetics and Developmental Biology, Chinese Academy of Sciences, Shijiazhuang, China; ^3^State Key Laboratory of Plant Cell and Chromosome Engineering, Chinese Academy of Sciences, Beijing, China; ^4^Genetic Improvement Centre of Agricultural and Forest Crops, College of Agriculture, Ludong University, Yantai, China; ^5^The Innovative Academy of Seed Design, Chinese Academy of Sciences, Beijing, China

**Keywords:** spike characteristics, low nitrogen tolerance, quantitative trait locus, conditional QTL mapping, wheat

## Abstract

Optimal spike characteristics are critical in improving the sink capacity and yield potential of wheat even in harsh environments. However, the genetic basis of their response to nitrogen deficiency is still unclear. In this study, quantitative trait loci (QTL) for six spike-related traits, including heading date (HD), spike length (SL), spikelet number (SN), spike compactness (SC), fertile spikelet number (FSN), and sterile spikelet number (SSN), were detected under two different nitrogen (N) supplies, based on a high-density genetic linkage map constructed by PCR markers, DArTs, and Affymetrix Wheat 660 K SNP chips. A total of 157 traditional QTLand 54 conditional loci were detected by inclusive composite interval mapping, among which three completely low N-stress induced QTL for SN and FSN (*qSn-1A.1, qFsn-1B*, and *qFsn-7D*) were found to maintain the desired spikelet fertility and kernel numbers even under N deficiency through pyramiding elite alleles. Twenty-eight stable QTL showing significant differencet in QTL detection model were found and seven genomic regions (R2D, R4A, R4B, R5A, R7A, R7B, and R7D) clustered by these stable QTL were highlighted. Among them, the effect of R4B on controlling spike characteristics might be contributed from *Rht-B1*. R7A harboring three major stable QTL (*qSn-7A.2, qSc-7A*, and *qFsn-7A.3*) might be one of the valuable candidate regions for further genetic improvement. In addition, the R7A was found to show syntenic with R7B, indicating the possibly exsting homoeologous candidate genes in both regions. The SNP markers involved with the above highlighted regions will eventually facilitate positional cloning or marker-assisted selection for the optimal spike characteristics under various N input conditions.

## Introduction

Wheat (*Triticum aestivum* L.) is one of the leading cereal crops worldwide, and is critical for global food security. The genetic improvement of three yield components, i.e., productive spikes per unit area, kernel number per spike (KN), and thousand kernel weight, contributing to the increase in wheat yield level and the alleviation of the food crisis in recent decades (Sayre et al., 1997; Ma et al., 2007; Deng et al., 2017). Of these three yield components, KN is directly determined by the spike characteristics (Cui et al., 2012). Therefore, researchers have been identifying important genes or quantitative trait loci (QTL) for spike characteristics in order to facilitate high-yield breeding programs (Zhai et al., 2016). For example, *Q, C*, and *S* are three major genes affecting spike characteristics and they are located on chromosomes 5A, 2D, and 3D (Johnson et al., 2008; Cui et al., 2012; Faris et al., 2014). In addition to controlling spike characteristics, these three genes present pleiotropy on the other traits. *Q* regulates the spike length, plant height (PH), and rachis fragility (Simons et al., 2006), whereas *S* can determine whether a spike has round seeds and glumes (Salina et al., 2000; Zhai et al., 2016). *C*, which is positioned in the interval of *Xgwm484*-*Xgwm358*-*Xcfd17*-*Xgwm539* on chromosomes 2DL (Johnson et al., 2008), accounts for the very dense “club” spike of club wheat (*Triticum compactum* Host) and has a pleiotropic effect on spike compactness, grain size, shape, and number (Johnson et al., 2008). However, these three genes are no longer the main breeding target for genetic improvement in modern wheat (Zhai et al., 2016), because most common wheat cultivars have identical genotypes, i.e., *QcS* (Faris et al., 2014). The heading date was another spike development-related trait and crucially affected the spikelet fertility, flower development, and grain filling. Three categories of genes, including vernalization-response genes (*Vrn*), photoperiod-response genes (*Ppd*), and earliness *per-se* genes (*Eps*), were well-known to control heading date (Yan et al., 2006; Cockram et al., 2007; Guedira et al., 2016) and were associated with spike development (Lewis et al., 2008; Zhang et al., 2014; Boden et al., 2015). In addition to these well-studied genes in wheat, there are other genes for spike characteristics being reported in barley, rice and maize, such as *MONOCULM* 1 (*MOC1*; Li et al., 2003; Zhang et al., 2015), *Six-rowed spike* genes (*Vrs*; Koppolu et al., 2013), *CONSTANS-*like gene (*CO*; Mulki and Von, 2016), etc. Among them, *CO* gene families play an important role in responding to the photoperiod in barley and rice (Griffiths et al., 2003), which affects the floret primordial loss and maximum floret number in wheat (Guo et al., 2016). *Vrs* is a regulator of spikelet fertility and controls spikelet determinacy in barley (Pourkheirandish et al., 2007; Koppolu et al., 2013). Although the contributions of these wheat orthologous genes on spike characteristics need to be further specified, it could be preliminarily evaluated at the QTL level.

As a complex and multicomponent trait, spike characteristic is comprehensively determined by a series of correlated traits, such as spike length, spikelet number, spike compactness etc. Major genes controlling these components usually present pleiotropy or linkage at the QTL level. Therefore, co-localized QTL regions are noteworthy when the putative candidate genes/loci for improving the integrated sink capacity are being examined. Previous studies have reported that numerous pleiotropic QTL clusters simultaneously affect multiple spike-related traits and involve major genes (Heidari et al., 2011; Cui et al., 2012; Zhai et al., 2016). For example, *Rht8* was found to co-localize with QTL for PH as well as QTL for the spike length, spikelet number, and spike compactness on 2DS (Ma et al., 2007; Cui et al., 2012; Xu et al., 2014; Wang et al., 2015; Zhai et al., 2016; Deng et al., 2017), and *Rht8c* was found to affect spike compactness by regulating the spike length (Kowalski et al., 2016). *Rht-B1* on 4BS, the famous green revolution gene, has a pleiotropic effect on not only the PH but also on the kernel weight, grain quality, seedling vigor and adaptability to harsh environments (McCartney et al., 2005; Zheng et al., 2010; Bai et al., 2013; Zhang et al., 2013b; Asif et al., 2015; Cui et al., 2016). However, only Heidari et al. (2011) reported that *Rht-B1* co-localized with QTL for spike compactness and its putative effect on other spike characteristics is rarely discussed.

In addition to be influenced by genetic background, spike characteristics are also influenced by many environmental factors, such as nitrogen (N) nutrition. Until now, numerous QTL for spike characteristics have been mapped on all 21 wheat chromosomes (Cui et al., 2012), but only a few studies have explored the QTL response to N application, which is useful for improving N use efficiency and yield potential in harsh nutrition environments. For example, Deng et al. (2017) detected several QTL for spike-related traits possibly induced by N application on chromosomes 2A, 2D, 4B,. 5A, 5B, 6A, and 7B under different levels of N treatments. However, few QTL that are sensitive to N deficiency, which are important for wheat adaption to low N tolerance, have been specified. Conditional QTL analysis is an efficient method to elucidate the influences of environmental factors on QTL expression based on trait values conditioned on different environments (Xu et al., 2014). Particularly, the QTL for drought tolerance in wheat and QTL for salt tolerance in maize were both successfully detected by excluding the influences of traits expressed under normal treatment (Zhang et al., 2013a; Cui et al., 2015).

Based on the high-density genetic linkage map using Wheat 660K SNPs (Cui et al., 2017), this study aimed to: (1) highlight the critical chromosomal regions harboring stable and pleiotropic QTL for spike characteristics including heading date (HD), spike length (SL), total spikelet number (SN), spike compactness (SC), fertile spikelet number (FSN), and sterile spikelet number (SSN), (2) specify the low N-stress induced QTL and identify the influence of N deficiency on the expression of these QTL under N treatments by traditional QTL analysis and conditional QTL analysis, and (3) preliminarily illuminate the possible pleiotropic effect of *Rht-B1* on spike characteristics.

## Materials and Methods

### Experimental Materials and Evaluation

A recombinant inbred line (RIL) population comprising 188 lines derived from a cross between Kenong 9204 (KN9204) and Jing 411 (J411) (represented by KJ-RILs) were used in this study (Fan et al., 2015; Cui et al., 2016, 2017; Zhang et al., 2017). The KJ-RILs and their parents were evaluated in four trials (year × location) as follows: 2011–2012 in Shijiazhuang (Trial 1: 37°53′N, 114°41′E, altitude 54 m); 2012–2013 in Shijiazhuang (Trial 2); 2012–2013 in Beijing (Trial 3: 40°06′N, 116°24′E, altitude 41 m); 2013–2014 in Shijiazhuang (Trial 4). In each trial, a low nitrogen (LN) treatment and high nitrogen (HN) treatment were applied for a total of eight environments, which were designated as T1LN, T1HN, T2LN, T2HN, T3LN, T3HN, T4LN, and T4HN. In the high N plots, N was applied as diamine phosphate at 180 kg•ha^−1^ before sowing and 225 kg•ha^−1^ of urea was applied at the elongation stage. In the LN plots, no N fertilizer (N-deficient) was applied during the growing period. The soil nitrogen contents at sowing in 0–40 cm soil depth in each plot were analyzed and shown in Table S1. The materials were planted in randomized complete blocks with two replications for each of the 8 environments. Each block contained two rows, 2 m long and 0.25 m apart and 40 seeds were evenly planted in each row. All nitrogen treatments, field arrangements, and experimental designs were previously described in detail previously (Fan et al., 2015; Zhang et al., 2017).

Six spike-related traits including HD, SL, SN, SC, SN, and SSN were evaluated in this study. HD was recorded as the day of the year in all environments when 50% of the plants in a plot were at Zadoks 59 growth stage (Guedira et al., 2016). At maturity, SL was measured from the base of the rachis to the tip of the terminal spikelet, excluding the awns. FSN and SSN were determined by counting the number of fertile and sterile spikelet per spike. SN was calculated by summing the values of FSN and SSN. SC was calculated by dividing the SL by SN. For each row, the main tillers of five plants were randomly chosen from the middle of the row to measure the phenotype.

### Data Analysis and QTL Mapping

The analysis of variance (ANOVA) and the phenotypic correlation coefficients were performed using SPSS 19.0 (SPSS, Chicago, MI, USA). The broad-sense heritability ($h_{B}^{2}$) was calculated using QGAStation 2.0 (http://ibi.zju.edu.cn/software/qga/v2.0/index_c.htm); the eight environments were regarded as replications and the genotype × environment interaction as the error term (Xu et al., 2014). Conditional analysis was performed to study the effects of LN-stress on QTL expression. All conditional phenotype value data were collected according to Zhu (1995) and Xu et al. (2014) using QGAStation 2.0. The conditional phenotype values (LN|HN) are the net genetic variation of trait values in LN independent of that in HN (Xu et al., 2014; Cui et al., 2015). Both the measured and the conditional phenotype values were used for QTL analysis which were designated as traditional QTL analysis and conditional QTL analysis, respectively.

Wheat660K, the Affymetrix® Axiom® Wheat660, was designed by the Chinese Academy of Agricultural Sciences and synthesized by Affymetrix. It is genome-specific array with high density and is highly efficient in a wide range of potential applications (http://wheat.pw.usda.gov/ggpages/topics/Wheat660_SNP_array_developed_by_CAAS.pdf). The high-density genetic map of KJ-RILs used to detect the QTL in this study was constructed by 119,001 SNP markers derived from the Wheat 660 K and 565 SSR, EST-SSR, ISSR, STS, SRAP, and DArT markers (Cui et al., 2017). This origin map contained a total of 119,566 loci. These 119,566 loci had 4,959 patterns of segregation in the 188 KJ-RILs, and 4,959 markers were chosen to represent each bin and were used for QTL mapping in this study. The present map has an average density of one marker per 0.89 cm and spans 4424.40 cm across 21 chromosomes. More information on this map is described by Cui et al. (2017).

PLN and PHN present the adjusted mean phenotypic value across four trials under the LN (T1LN, T2LN, T3LN, and T4LN) and HN (T1HN, T2HN, T3HN, and T4HN) treatments, respectively. The traditional phenotype value of T1LN, T1HN, T2LN, T2HN, T3LN, T3HN, T4LN, T4HN, PLN, and PHN, and the conditional phenotype values (LN|HN) in T1, T2, T3, T4, and P (the adjusted mean conditional phenotypic value (LN|HN) across four trials) were used to detect QTL by inclusive composite interval mapping (ICIM) performed by IciMapping 4.1 (Li et al., 2007; freely download from http://www.isbreeding.net/). The walking speed chosen for all QTL was 1.0 cm, and the *P*-value inclusion threshold was 0.001. The LOD scores of 3.0 were used to detect and declare the presence of a putative QTL (Zhang et al., 2017). Furthermore, a QTL with an LOD value >5.0 and a phenotypic variance contribution >10% (on average) was defined as the major QTL; a QTL with an LOD value >3.0 but <5.0 and a phenotypic variance contribution <10% (on average) was defined as a moderate QTL; a QTL detected only under HN or LN conditions was defined as the HN- or LN- specific QTL (Fan et al., 2015; Zhang et al., 2017).

Moreover, except for the single-environment QTL detection based on ICIM, multi-environment QTL analysis was conducted using GenStat 19.0 across all environments to verify the traditional QTL identified in the individual environment and to evaluate the QTL × environment interaction effects (Payne et al., 2013). A best variance-covariance model was selected based on the Schwarz Information Criterion for phenotype data from a set of multi-environment experiments (Schwarz, 1978; Malosetti et al., 2013). The QTL and QTL × environment interaction effects were determined by testing the significance of environment-specific deviations from the main environmental effects using a Wald test (Verbeke and Molenberghs, 2000). The degree of phenotypic variation explained by an individual QTL was calculated as described by Asfaw et al. (2012). Finally, a QTL that could be detected by both single-environment and multi–environment QTL detection was defined as a stable QTL, considering its significant stability even based on different detection models. For a given trait, QTL and loci with overlapping CIs (LOD ≥2) were assumed identical (Zhang et al., 2017). Among different traits, QTL sharing flanking markers were considered to be a “cosegregation” QTL region (Cui et al., 2016).

The reported spike related genes/loci and their genetic positions were obtained in the literature; these included, including *Photoperiod-1* (*Ppd-A1, Ppd-B1*, and *Ppd-D1*) (Boden et al., 2015), the vernalization responsive genes (*Vrn-1A, Vrn-1B, Vrn-1D, Vrn2*, and *Vrn3*) (Yan et al., 2004, 2006; Dubcovsky et al., 2006), the earliness *per se* locus *Eps-Am1* (Lewis et al., 2008), the spike-compacting related locus (*C*) (Johnson et al., 2008), the floral organ development related gene *TaANT* (Zhang et al., 2014), the semi-dwarfing genes *Rht-B1* and *Rht8* (Ellis et al., 2007; Zhang et al., 2013b, 2017), and the wheat orthologous genes corresponding to *Six-rowed spike 1* (*Vrs1*) (Komatsuda and Tanno, 2004), *CONSTANS*, and *MOC1* (Campoli et al., 2012). The Chinese Spring genome assembly from the International Wheat Genome Sequencing Consortium (IWGSC) Reference Sequence v1.0 was used as the reference genome. The available sequences of these reported spike-related genes/loci were retrieved in the Gene Bank through the NCBI website (http://www.ncbi.nlm.nih.gov/) and used as queries in a BLAST search in the IWGSC website (https://urgi.versailles.inra.fr/blast_iwgsc/blast.php) to obtain their physical position. By comparing their position with the highlighted regions in this study, the possible candidate genes were preliminarily screened.

## Results

### Phenotypic Data and Correlation Analyses

The genetic variation of six investigated traits in the KJ-RILs is shown in Table S2, which presented the ANOVA results for the phenotypic data. ANOVA showed that genotype, environment and genotype × environment had significant effects on HD, SL, SN, SC, FSN, and SSN. KN9204 has an earlier HD, shorter SL, fewer SN and FSN, more SSN, and tighter SC than those of J411 (Table S3). In the KJ-RIL population, the six traits exhibited approximately continuous variation in each treatment in four trials. Transgressive segregation was observed in both high and low sides in this population (Table S3), indicating that alleles with positive effects were contributed by both parents. Additionally, the absolute values of skewness and kurtosis were almost <1 (Table S3), indicating that the phenotypic data were approximately normally distributed in this population. Heritability ranged from 50.30 to 77.06% (Table S3). A significant positive correlation was observed between SN and the other investigated traits (Table S4). The SL was significantly negatively correlated with SC, whereas it was positively correlated with the other examined traits (Table S4). FSN was significantly negatively correlated with SSN while positively correlated with the other investigated traits (Table S4). The most significant correlation coefficient was observed between SL and SN (0.888) (Table S4).

### Traditional QTL Analysis Under Different Nitrogen Treatments

For single-environment QTL detection, a total of 157 QTL for the six spike characteristics (Table S5), were detected by IciMapping 4.1, distributed on all 21 chromosomes with QTL phenotypic variations ranging from 1.55 to 26.26% and LOD value of 3.01–22.22. Among these 157 QTL, 41 could be detected in different trials, and 11 QTL were major QTL (Table S5).

Additionally, for multi-environment QTL detection, 30 loci were identified using GenStat 19.0 (Table S6). Twenty-eight loci show significant interaction effect with environment (Table S6). The The CIs of 28 loci overlapped with the corresponding single-environment QTL listed in Table 1. Thus, these 28 QTL were considered stable QTL, and 78.57% of them (22 QTL) could be detected in different trials based on ICIM (Table 1). It is notable that all the 11 major QTL could be repeatedly detected by GenStat 19.0 and were stable QTL (Table 1, Tables S5, S6).

**Table 1:** The stable QTL detected in this study.

**Traits**	**QTL^**a**^**	**Left CI**	**Right CI**	**Environments**	**Mean of LOD**	**Mean of PVE (%)**	**Mean of additive effect**
HD (days)	*qHd-1B.1*	14.50	17.50	T2HN	6.08	8.20	0.37
	***qHd-2D.1***	88.50	90.50	T1HN, T2LN, T2HN, T3LN, T3HN, T4LN, T4HN, PLN, PHN	12.22	18.39	−0.54
	***qHd-4B.1***	35.50	46.00	T1HN, T2LN, T2HN, T3LN, T3HN, T4LN, T4HN, PLN, PHN	8.32	14.03	0.64
SL (cm)	***qSl-2D.1***	125.50	131.50	T1LN, T1HN, T3HN, PHN	10.91	13.20	−0.29
	***qSl-5A.3***	64.50	67.50	T1LN, T1HN, T2LN, T2HN, T3LN, T3HN, T4LN, T4HN, PLN, PHN	16.02	20.56	−0.27
	*qSl-7A.2*	152.50	154.50	T2LN	5.48	5.19	−0.17
SN	*qSn-1A.1*	21.50	22.50	T2LN	4.28	3.61	−0.24
	*qSn-4A*	151.50	156.50	T1LN, T2LN	3.44	4.58	−0.25
	*qSn-5A.2*	57.50	62.50	T2LN, T3HN, PLN	7.64	6.29	−0.29
	***qSn-7A.2***	160.50	161.50	T1LN, T1HN, T2LN, T2HN, T3LN, T3HN, T4LN, T4HN, PLN, PHN	22.22	26.26	−0.71
	*qSn-7B.2*	110.50	111.50	T2LN, T3LN, T3HN	6.55	4.2	−0.25
	*qSn-7D*	166.50	170.50	T1LN, T1HN, T2HN, T3LN, T3HN, PLN, PHN	9.89	9.12	0.43
SC	*qSc-2B.1*	44.50	57.50	T1LN, T1HN, T2HN, T3LN, T3HN, T4LN, T4HN, PLN, PHN	4.35	5.09	−0.06
	*qSc-2D*	89.50	90.50	T4HN	5.56	7.54	−0.06
	***qSc-5A.2***	62.50	67.50	T1LN, T1HN, T2LN, T2HN, T3LN, T3HN, T4LN, T4HN, PLN, PHN	13.39	19.93	0.14
	***qSc-7A***	160.50	161.50	T1LN, T1HN, T2LN, T2HN, T3LN, T3HN, T4LN, T4HN, PLN, PHN	9.30	13.97	−0.09
	*qSc-7B*	108.50	115.50	T1LN, T1HN, T2LN, T3LN, T4HN, PLN	5.58	8.22	−0.06
FSN	*qFsn-1B.1*	9.50	12.50	T1LN, PLN	4.46	9.47	0.36
	*qFsn-3A.1*	141.50	144.50	T1LN, T1HN, T2HN, T3HN, T4LN, T4HN, PHN	5.12	6.59	0.35
	*qFsn-4A*	150.50	156.50	T1LN, T2LN, T3LN, T4HN, PLN	4.78	5.75	−0.28
	***qFsn-4B.2***	39.50	43.50	T1LN, T1HN, T3LN, T4LN, T4HN, PLN, PHN	11.14	13.64	0.55
	***qFsn-7A.3***	154.5	161.5	T1LN, T1HN, T2LN, T2HN, T3LN, T3HN, T4LN, PLN, PHN	19.53	23.45	−0.61
	*qFsn-7D*	170.50	171.50	T2LN, T3LN, PLN	4.53	4.79	0.25
SSN	*qSsn-1B.1*	1.50	3.50	T2LN, T4LN, T4HN	4.46	12.92	0.36
	***qSsn-4B.3***	40.50	44.50	T3LN, T3HN, T4LN, T4HN, PLN	8.32	10.17	−0.28
	***qSsn-5D.1***	0	5.50	T1LN, T1HN, T2LN, T2HN, T3LN, T3HN, T4HN, PLN, PHN	8.75	11.22	0.24
	*qSsn-6B*	94.50	95.50	T2LN, PLN	6.96	8.16	0.13
	*qSsn-7D.2*	162.5	171.5	T1LN, T1HN, T2LN, T2HN, T3LN, T3HN, T4LN, T4HN, PLN, PHN	5.26	7.29	0.19

*The positive value of additive effect indicates that the KN9204 allele increases the corresponding traits. The negative value indicates that the J411 allele increases the corresponding traits*.

a *The QTL in bold are the major QTL. The underlined QTL are the QTL which could be detected in different trials by IciMapping 4.1*.

### The Traditional QTL for HD

Twenty-five traditional QTL for HD were identified by single-environment QTL detection, and eight and 12 of them were LN- and HN-specific QTL, respectively (Table S5). qHd-1B.1, qHd-2D.1, and qHd-4B.1 could be repeatedly detected by multi-environment QTL detection (Table S6) and thus were stable QTL (Table 1). Additionally, qHd-2D.1 and qHd-4B.1 were major and stable QTL (defined as major stable QTL) detected under nine datasets except T1LN, explaining 8.58–27.01% and 3.36–22.29% of the HD variation, respectively. KN9204-derived alleles could advance the HD at the locus of qHd-2D.1 while delay HD at the locus of Hd-4B.1 (Table S5).

### The Traditional QTL for SL

A total of 26 QTL associated with SL were detected based on ICIM, thirteen and six of them were LN- and HN-specific QTL, respectively (Table S5). qSl-2D.1, qSl-5A.3, and qSl-7A.2 were three stable QTL through verifying by GenStat software (Table 1, Table S6). The major QTL on 2D (qSl-2D.1) was significant in T1LN, T1HN, T3HN, and PHN, explaining 7.99–16.58% of the phenotypic variation. The other major QTL on 5A (qSl-5A.3) were detected in ten datasets and explained 13.09–30.43% of the SL variation. The additive effects of the two major QTL showed that the negative alleles (shortening SL) originated from the parent with the shorter SL, ie., KN9204 (Table S5).

### The Traditional QTL for SN

For single-envrionment QTL detection, thirty-one QTL were identified, and among them, nine and 14 were LN- and HN-specific QTL (Table S5), respectively. Six QTL (qSn-1A.1, qSn-4A, qSn-5A.2, qSn-7A.2, qSn-7B.2, and qSn-7D) show overlap of the CIs with the loci detected by GenStat software (Table S6) and were stable QTL (Table 1). The only major QTL on 7A (qSn-7A.2) was observed under both LN and HN treatments in all trials, which had LOD value of 4.99–39.13 and explained 9.80–43.37 % of the SN variation, with KN9204-derived allele decreasing SN (Table S5).

### The Traditional QTL for SC

Twenty-one QTL for SC were detected in individual environments based on ICIM method, eleven and five of which were LN- and HN-specific QTL, respectively (Table S5). Five stable QTL (qSc-2B.1, qSc-2D, qSc-5A.2, qSc-7A, and qSc-7B) were significant by multi-environment QTL detection (Table 1, Table S6). Among them, qSc-5A.2 and qSc-7A were two major QTL explaining 13.12–28.38% and 4.87–17.22%of the SC variation, with the LOD value of 7.37–20.40 and 3.67–15.04, respectively, in all datasets. KN9204 conferred an effect for an increased SC at the former locus but a decreased SC at the latter one. Additionally, a moderate but stable QTL (defined as moderate stable QTL) on 2B (qSc-2B.1) could be repeatedly detected in nine datasets, except for T2LN (Table S5).

### The Traditional QTL for FSN

Twenty-eight QTL for FSN were mapped by single-environment QTL detection. Twelve and eight of them were LN- and HN-specific QTL, respectively (Table S5). Six stable QTL, including qFsn-1B.1, qFsn-3A.1, qFsn-4A, qFsn-4B.2, qFsn-7A.3, and qFsn-7D were repeatedly identified by multi-environment QTL detection (Table 1, Table S6). qFsn-4B.2 was a major stable QTL which had LOD value of 5.13–18.36 and explained 6.30–24.43% of the phenotypic variation in T1LN, T1HN, T3LN, T4LN, T4HN, PLN, and PHN. qFsn-7A.3 was the other major stable QTL expressed in nine datasets except T4HN, with LOD value of 4.61–36.38 and PVE of 9.11–36.41%. An additive effect of qFsn-4B.2 showed that the superior allele originated from KN9204, while the superior allele of qFsn-7A.3 came from J411. Additionally, qFsn-3A.1 was found to be a moderate stable QTL and was expressed in seven datasets, except for T2LN, T3LN, and PLN (Table S5).

### The Traditional QTL for SSN

In single-envrionment QTL detection, twenty-six QTL for SSN were identified, nine and 9 of which were LN- and HN-specific QTL, respectively (Table S5). qSsn-1B.1, qSsn-4B.3, qSsn-5D.1, qSsn-6B, and qSsn-7D.2 were five stable QTL, as verified by GenStat software (Table 1, Table S6). Two major stable QTL (qSsn-4B.3 and qSsn-5D.1), show significance in five and ten datasets, respectively. qSsn-4B.3 and qSsn-5D.1 explained 6.80–11.38% and 9.14–17.01% of the phenotypic variation, with LOD values of 5.25–9.70 and 6.16–12.03, respectively. A moderately stable QTL expressed in all the ten datasets was detected on 7D (qSsn-7D.2) (Table S5).

### Conditional QTL Analysis With Respect to LN-Stess-Inducible QTL

By comparing the additive effects of the traditional QTL detection (Table S5) and conditional QTL detection based on the trait values of LN conditioned on that of HN (LN|HN) (Table S7), the effects of LN-stress on QTL expression for spike characteristics could be evaluated (Xu et al., 2014; Cui et al., 2015). A total of 54 loci were significant in the conditional QTL analysis using IciMapping 4.1 (Table S7). They explained 3.17–14.70% of the phenotypic variation and showed LOD values of 3.00–8.02. Only five loci associated with SN, SC, and FSN had main effects (average LOD > 5 and average PVE > 10 %), and four loci for SN, SC, and FSN were repeadly detected in different trials (Table S7). Among the conditional loci, 21 were also identified in traditional QTL analysis (Table S5) and listed in Table 2, while the other 33 were newly detected (Table S7).

**Table 2:** The loci for spike characteristics significant using both traditional and conditional QTL mapping.

**Traits**	**Loci detected by conditional analysis^**a**^**	**The corresponding QTL^**b**^**	**Additive effect In Traditional QTL mapping**	**Additive effect In Conditional QTL mapping**
HD (days)	Locus 3	*qHd-7B.1*	−0.46	−0.19
SL (cm)	Locus 6	*qSl-2B*	0.14	0.10
	Locus 7	*qSl-2D.1*	−0.29	−0.12
	Locus 14	*qSl-7A.1*	0.15	−0.10
SN	**Locus 15**	qSn-1A.1^*^	−0.24	−0.25
	*Locus 20*	*qSn-7A.2*	−0.71	−0.20
	Locus 21	*qSn-7B.2*	−0.25	−0.17
SC	Locus 23	*qSc-2A.1*	0.04	0.03
	Locus 26	*qSc-4B.2*	0.07	0.16
	***Locus 27***	*qSc-5A.2*	0.14	0.09
	**Locus 29**	*qSc-7A*	−0.09	−0.04
	Locus 30	*qSc-7B*	−0.06	−0.11
FSN	**Locus 32**	*qFsn-1A*	−0.22	−0.29
	Locus 33	qFsn-1B.1^*^	0.36	0.35
	Locus 34	*qFsn-2B*	−0.24	−0.19
	**Locus 41**	*qFsn-7A.3*	−0.61	−0.23
	*Locus 43*	qFsn-7D^*^	0.25	0.23
SSN	Locus 44	*qSsn-1B.1*	0.36	0.15
	Locus 49	***qSsn-4B.3***	−0.28	−0.20
	Locus 51	*qSsn-5D.1*	0.24	0.12
	Locus 52	*qSsn-6B*	0.13	0.09

*The positive value of the additive effect indicates that the KN9204 allele increases the corresponding traits. The negative value indicates that the J411 allele increases the corresponding traits*.

a *The underlined loci indicate that they can be repeatedly detected in different trials by conditional QTL analysis; the locus in bold indicates it is with main effect*.

b *The underlined QTL were QTL that were detected in different datasets based on the ICIM method*.

* *Indicates that the QTL are completely LN-stress induced QTL in which is the absolute values reduces or increases <10% compared to the corresponding traditional QTL, respectively (Fan et al., 2015)*.

By comparing the additive effects of traditional analysis and conditional analysis based on trait values of LN conditioned on that of HN, the effects of N deficiency on the QTL expression of related traits could be evaluated. For example, if a conditional locus conditioned on HN has a similar or greatly different effect to its traditional QTL, demonstrating that the QTL is completely or partially contributed by LN stress, whereas when an traditional QTL cannot be detected again when conditioned on HN, the QTL is considered to mainly controlled by N supplementation. In detail, one, four, two, five, five, and four traditional QTL for HD, SL, SN, SC, FSN, and SSN could also be detected by conditional QTL analysis, respectively (Table 2, Tables S5, S7), and fourteen QTL could also be detected by GenStat software (Table S6). By comparing the additive effects of traditional QTL mapping results, three and eighteen corresponding conditional loci individually showed similar or greatly different effect values, implying that their expression were completely or partially affected by LN-stress, respectively. Among them, three loci (corresponding to qSn-1A.1, qFsn-1B.1, and qFsn-7D) were detected only under LN treatment by traditional QTL analysis and showed a significant interaction with environment, and all three loci were found to have the similar additive affects using both conditional and traditional QTL mapping (Table 2); thus, they were considered to be the completely LN-stress-induced QTL. Furthermore, qFsn-1B.1 and qFsn-7D were stable QTL (Tables 1, 2).

### Stable QTL Regions for Spike Characteristics

**Figure 1:**
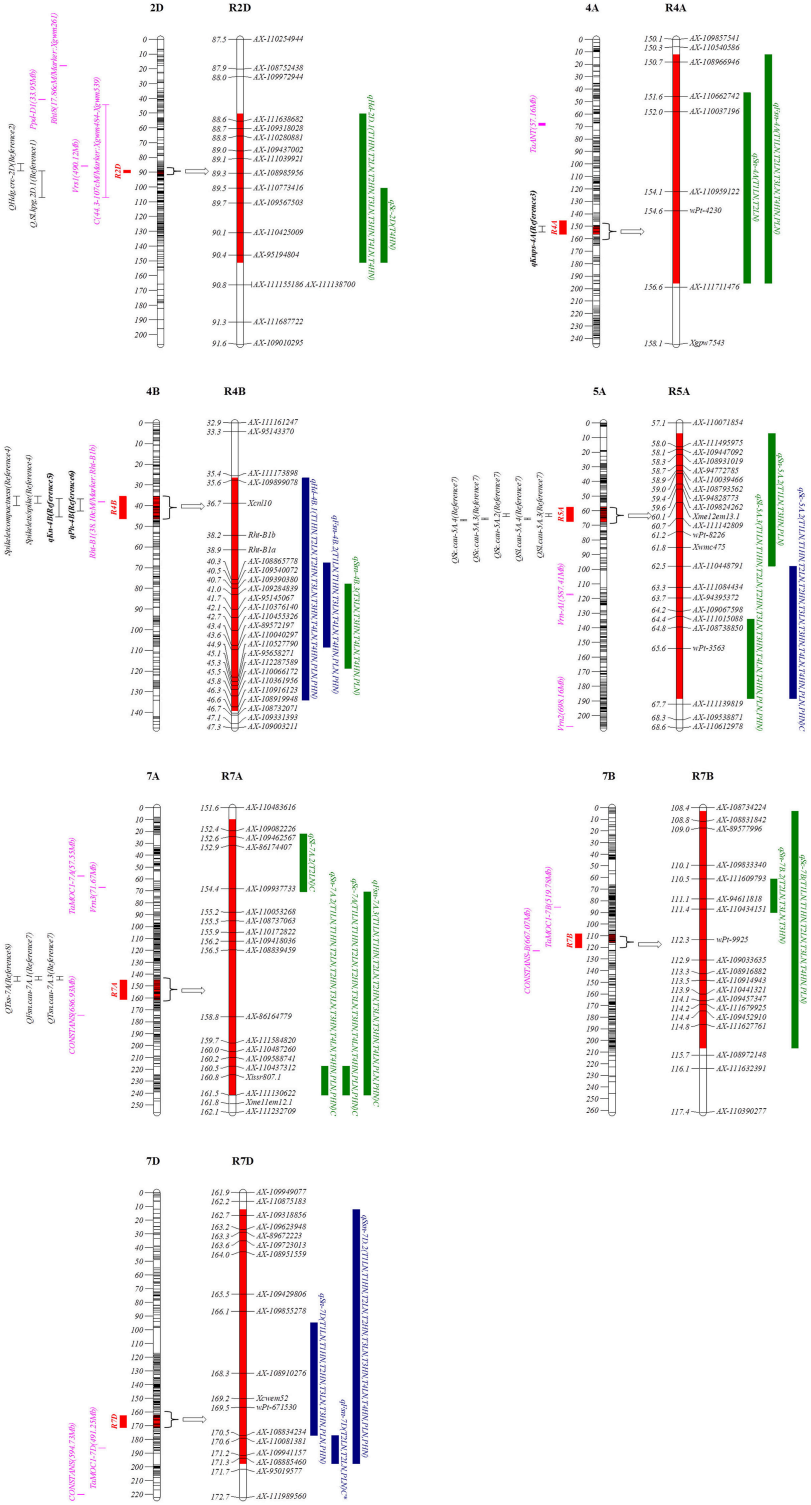
Chromosomal locations of seven highlighted genomic regions for spike characteristics in this study. The red intervals on the chromosomes and regions represent the LOD-3 confidence interval of the location of each genomic region. To the left of the chromosomes: the centiMorgan (cm) interval is shown; the red rectangles represent each QTL region; the purple lines represent the putative location of the screened genes associated with spike-related traits; the black intervals represent the coincident QTL in previous reports (Reference 1 to Reference 11), and among them, three QTL in bold (qKnps-4A, qPh-4B, and qKn-4B) were detected using the same population of this study (KJ-RILs). On the right of each chromosomal region: the blue and green rectangles represent the QTL with positive alleles from KN9204 and J411, respectively; the letter C after parentheses represents the expression of QTL induced by N deficiency, and among them, C^*^ represents the QTL (qFsn-7D) is a completely LN-stress induced QTL. Reference 1: (Nishijima et al., 2017); Reference 2: (Cuthbert et al., 2008); Reference 3: (Cui et al., 2017); Reference 4: (Heidari et al., 2011); Reference 5: (Zhang et al., 2017); Reference 6: (Fan et al., 2015); Reference 7: (Zhai et al., 2016); Reference 8: (Xu et al., 2014).

Based on the stable QTL detected in this study (Table 1), seven genomic regions containing 19 stable QTL for different traits were highlighted (Table 3; Figure 1). These clustered stable QTL shared confidence intervals and thus were indicative of potential pleiotropic effect on the corresponding traits. Among them, R7D harbored KN9204-derived alleles for simultaneously increasing the corresponding traits, while four regions (R2D, R4A, R7A, and R7B) harbored the J411-derived alleles that contributed to the increasing alleles. Four regions (R2D, R4B, R5A, and R7A) contained major stable QTL (Table 3; Figure 1).

**Table 3:** Seven genomic regions harboring the stable QTL in this study.

**Genomic regions**	**Coincident genes**	**Chr**.	**Right markers**	**Left markers**	**Interval (cM)**	**Physical range (bp)**	**QTL detected in this studies^**a**^**	**Reported QTL for spike related traits**
R2D	*C; Vrs1*	2DL	*AX-109972944*	*AX-111155186*	88.50–90.50	347812780: 419435778	***qHd-2D.1 (9, –)***, *qSc-2D (1, –)*	HD (Cuthbert et al., 2008; Nishijima et al., 2017)
R4A		4AL	*AX-110540586*	*AX-111711476*	150.50–156.50	669581472: 680398669	*qSn-4A (2, –)*, *qFsn-4A (5, –)*	
R4B	*Rht-B1*	4BS	*AX-111173898*	*AX-108919948*	35.50–46.00	27521871: 88377629	***qHd-4B.1 (9, +)***, ***qFsn-4B.2 (7, +)***, ***qSsn-4B.3 (5, –)***	SC (Heidari et al., 2011)
R5A		5AL	*AX-110071854*	*AX-111139819*	57.50–67.50	478645804: 541292147	***qSl-5A.3 (10, –)***, *qSn-5A.2 (3, –)*, ***qSc-5A.2 (10, +)***	SL (Zhai et al., 2016), SC (Zhai et al., 2016)
R7A		7AL	*AX-110487560*	*AX-110437312*	152.00–161.50	660452399: 675326249	*qSl-7A.2 (1. –)*, ***qSn-7A.2 (10, –)***, ***qSc-7A (10, –)***, *qFsn-7A.3 (9, –)*	SN (Xu et al., 2014; Zhai et al., 2016); FSN (Zhai et al., 2016)
R7B	*CONSTANS*	7BL	*AX-108734224*	*AX-108972148*	108.50–115.50	639497944: 656427446	*qSn-7B.2 (3, –)*, *qSc-7B (6, –)*	SN (Liu et al., 2018)
R7D		7DS	*AX-110875183*	*AX-95019577*	162.50–171.50	168771913: 218177431	*qSn-7D (7, +)*, *qFsn-7D (3, +)*, *qSsn-7D.2 (10, +)*	

a *The QTL in bold are the major QTL. The underlined QTL are the QTL which could be repeatedly detected in different trials by IciMapping 4.1. The number in the parentheses indicate the sum of single-environment datasets in which the corresponding QTL are significant. The “+” in the parentheses indicates that KN9204 allele increases the corresponding traits. The “–” in the parenthesis indicates that J411 allele increases the corresponding traits*.

The approximate physical position was searched by the flanking bin marker as a BLAST query. By comparing the position of the reported genes/QTL controlling the spike characteristics, the putative corresponding genes, and coincident QTL were screened. Until now, the candidate region of the spike-compacting locus *C* (Johnson et al., 2008) was found to cover R2D (Table 3; Figure 1). Furthermore, in R7B, the wheat orthologous of the barley gene *CONSTANS* (Campoli et al., 2012) was identified (Table 3; Figure 1). R4B, harboring two major stable QTL (*qHd-4B.1* and *qFsn-4B.2*) and a moderate stable QTL (*qSsn-4B.3*), just covered the semi-dwarfing gene *Rht-B1* (Table 3; Figure 1), indicating that *Rht-B1* might have a pleiotropic effect on the spike characteristics.

### Validation of the Consequences of *Rht-B1*

To preliminarily confer the pleiotropic effect of *Rht-B1* on the spike characteristics, genotyping of the diagnostic marker for the *Rht-B1* alleles (*Rht-B1a* and *Rht-B1b*) (Zhang et al., 2017) was used to group the KJ-RILs. Of the 188 KJ-RILs, 87, and 95 RILs were consistent with the genotypes of the alleles from the shorter parent KN9204 (with *Rht-B1b*) and taller parent J411 (with *Rht-B1a*), respectively (Table 4). The average phenotypic value under both the LN and HN treatments were used to identify differences in corresponding traits between *Rht-B1b* and *Rht-B1a*. The results indicated that, regardless of N treatments, the *Rht-B1b* was associated with the significant reduction in PH and SSN, and a remarkable increase in KN, HD, SN, and FSN, but no significant effect was detectedin the SL and SC (Table 4), which was coincident with the QTL mapping results (Table 3, Table S5).

**Table 4:** Validation of the putative pleiotropy of *Rht-B1b* on spike characteristics and kernel number.

**Genotypes**	***N* Treatments**	**PH**	**KN**	**HD**	**SL**	**SN**	**SC**	**FSN**	**SSN**
*Rht-B1b*	LN	68.29 ± 6.17^***^	42.12 ± 4.36^**^	210.81 ± 1.10^***^	7.96 ± 0.61	18.61 ± 1.04^*^	2.37 ± 0.18	16.92 ± 0.99^***^	1.69 ± 0.42^***^
*N* = 87	HN	72.29 ± 6.75^***^	41.92 ± 5.12^**^	211.87 ± 1.12^***^	8.38 ± 0.66	19.06 ± 1.05^**^	2.32 ± 0.18	17.22 ± 1.05^***^	1.84 ± 0.49^***^
*Rht-B1a*	LN	75.25 ± 7.37	40.20 ± 4.33	210.04 ± 1.24	7.93 ± 0.69	18.23 ± 1.03	2.34 ± 0.20	16.28 ± 0.91	1.95 ± 0.50
*N* = 95	HN	81.23 ± 8.13	38.97 ± 4.29	211.05 ± 1.30	8.23 ± 0.71	18.66 ± 0.95	2.31 ± 0.19	16.48 ± 0.85	2.17 ± 0.53

*The significant difference noted indicates that significant differences were detected between two genotypes containing Rht-B1b and Rht-B1a, respectively, under the LN and HN treatments*.

* *Indicates significance at the level of 0.05*.

** *Indicates significance at the level of 0.01*.

*** *Indicates significance at the level of 0.001*.

## Discussion

### QTL for Spike Characteristics Show Sensitivity to Nitrogen Supply

N is an important environmental factor determining spike and yield formation. Spike development was positively correlated with the N fertilizer application, which could optimize the kernel number per spike with increased spikelet number, spike N content accumulation and spikelet fertility and, ultimately, affect the yield (Corke and Atsmon, 1988; Demotes-Mainard et al., 1999). Thus, exploring the genetic basis of spike formation under different N conditions could excavate the useful nitrogen response loci to high nitrogen-use-efficiency and high yield breeding. The QTL identified under a specific nitrogen treatment are probably involved in the adaptation to nitrogen fertilizer management (Laperche et al., 2007). In this study, of the 157 detected QTL, 73.89% (62 LN-specific QTL and 54 HN-specific QTL) were N-supply-specific QTL (Table S5), thereby suggesting that the genetic basis of the spike characteristics was sensitive to the N treatment and thus could provide an appropriate and timely response strategy when adapting to variable N fertilizer level in the field, possibly through motivating different genetic regulatory network. However, we noticed only 9 N-supply-specific QTL (6 LN-specific QTL and 3 HN-specific QTL) stably show significancy in different trials, and none of them could express phenotypic variation >10% (Table S5), indicating that the regulatory network for N supply might be synergistically controlled by multiple moderately inducible genes. Therefore, considering the common sensitivity, pyramiding multiple loci, especially the stable loci, might be an efficient copying strategy to specific N supply conditions.

In addition to the N-supply-specific QTLs, eleven major stable QTLs could be identified under both the LN and HN treatments (Table S5). This result confirmed that the expression of these QTLs was stable and more unsusceptible to nitrogen supply, suggesting that their close linkage markers are of value in selecting and breeding optimal spike characteristics, regardless of nitrogen constraints.

### LN-Stress-Induced Loci for Spike Characteristics Were Efficiently Identified When Conditioned on HN

A boost in crop productivity was observed in recent decades through the global use of nitrogen fertilizers. However, the consequent nitrogen pollution prompted modern breeders to prefer to develop LN-tolerant cultivars adapted to environmentally friendly, low-input agricultural systems. The interactive relationship between N application and spike development was demonstrated by QTL mapping in previous studies (Xu et al., 2014; Deng et al., 2017). However, few studies specifically examined the influence of LN-stress on the expression of QTL for spike characteristics. These LN-stress induced QTL might be more valuable for improve the ability to maintain desired spike type in LN input agricultural practices. In this study, to further unravel loci sensitive to N deficiency and specify their responsiveness to LN stress, conditional QTL analysis was conducted and 54 LN-stress induced loci were detected (Table S7). Of them, 33 loci (61.11 %) could only be detected when conditioned on HN, indicating that most LN-stress induced loci were suppressed by N application (Table S7), which is consist with the previous conclusion (Zhang et al., 2013a; Cui et al., 2015). The different responsiveness of these LN-induced loci was also evaluated by comparing their additive effect detected between traditional and conditional QTL mapping (Xu et al., 2014). As a result, three completely LN stress induced QTL for SN and FSN (*qSn-1A.1, qFsn-1B.1*, and *qFsn-7D*) (Table 2) were considered critical for LN tolerance. Pyramiding the elite alleles of the above three QTL might be an optimal approach in wheat molecular breeding programs to acquire desired spikelet number and spikelet fertility under N deficiency. To verify this deduction, the SN, FSN, and KN was further investigated by pyramiding effect analysis. The results (Table S8) demonstrated that SN and FSN were significantly higher in the Type 1 genotype (which contained all three elite alleles) than in the Type 8 genotype (which contained none of the elite allele) under both LN and HN conditions and that KN was also increased in the favorite genotype (Type 1). Interestingly, all differences between HN and LN (HN-LN) in SN, FSN, and KN were remarkably decreased through pyramiding of the three elite alleles (Type 1), indicating that these three completely LN-induced QTL provided certain genetic support to maintain yield potential, and thus their linked marker could be used in breeding LN tolerant wheat. In addition, *qSn-7A.2*, a phosphorus (P)-contributed QTL detected by Xu et al. (2014), was found to be partially induced by N deficiency in different trials in this study (Table 2), suggesting that the antagonistic effect of N and P on its expression could be futher dissected.

### Comparison of the Present Findings With Previous Studies

The optimization of multiple spike characteristics can efficiently enhance the integrated sink capacity and yield potential. Nineteen stable QTL were clustered into seven pleiotropic genomic regions (Table 3; Figure 1). According to the common PCR-markers and the physical position of the SNP markers, five regions on chromosomes 2DL, 4BS, 5AL, 7AL, and 7BL were involved with the coincident QTL that were previously reported. For example, the CI of R2D was distal to *Ppd-D1* and *Rht8* were genes on chromosome 2D that were two well known to control HD and SC (Boden et al., 2015; Kowalski et al., 2016). However, the wheat orthologous gene of *HvVrs1*, which determined spike morphology (Komatsuda and Tanno, 2004), was located nearby R2D (around 85.13 cm). And the CI of the *C* locus, which was previously mapped in the interval of *Xgwm484*-*Xgwm539* by Johnson et al. (2008), also covered the R2D in this map. Thus, *C* and *Vrs1* have greater possibility of contributing to *qSc-2D* and *qSsn-2D* in R2D, rather than *Ppd-D1* and *Rht8*. Considering that the major stable QTL *qHd-2D.2* could also express in different genetic backgrounds (Li et al., 2002; Cuthbert et al., 2008), R2D, which affect not only the spike morphology but also the heading date, was deduced to harbor pleiotropic or clustered genes, probably involved with *C* and *Vrs*, to some extent supporting the significant correlation between HD and most other spike characteristics in this study (Table S4).

In R5A (Table 3, Figure 1), the major QTL *qSl-5A.3* and its colocalized major QTL for SC (*qSc-5A.2*), the J411-derived alleles could increase SL but decrease SC with remarkable stability in both the KN9204/J411 and Y8679/J411 RIL population (Zhai et al., 2016), possibly providing the critical stable genetic basis and thereby accounting for the significant negative correlation between SL and SC (Table S4). Moreover, the CI of R5A was far from *Vrn-A1*, which is in accordance with the mapping results by Zhai et al. (2016).

**Figure 2:**
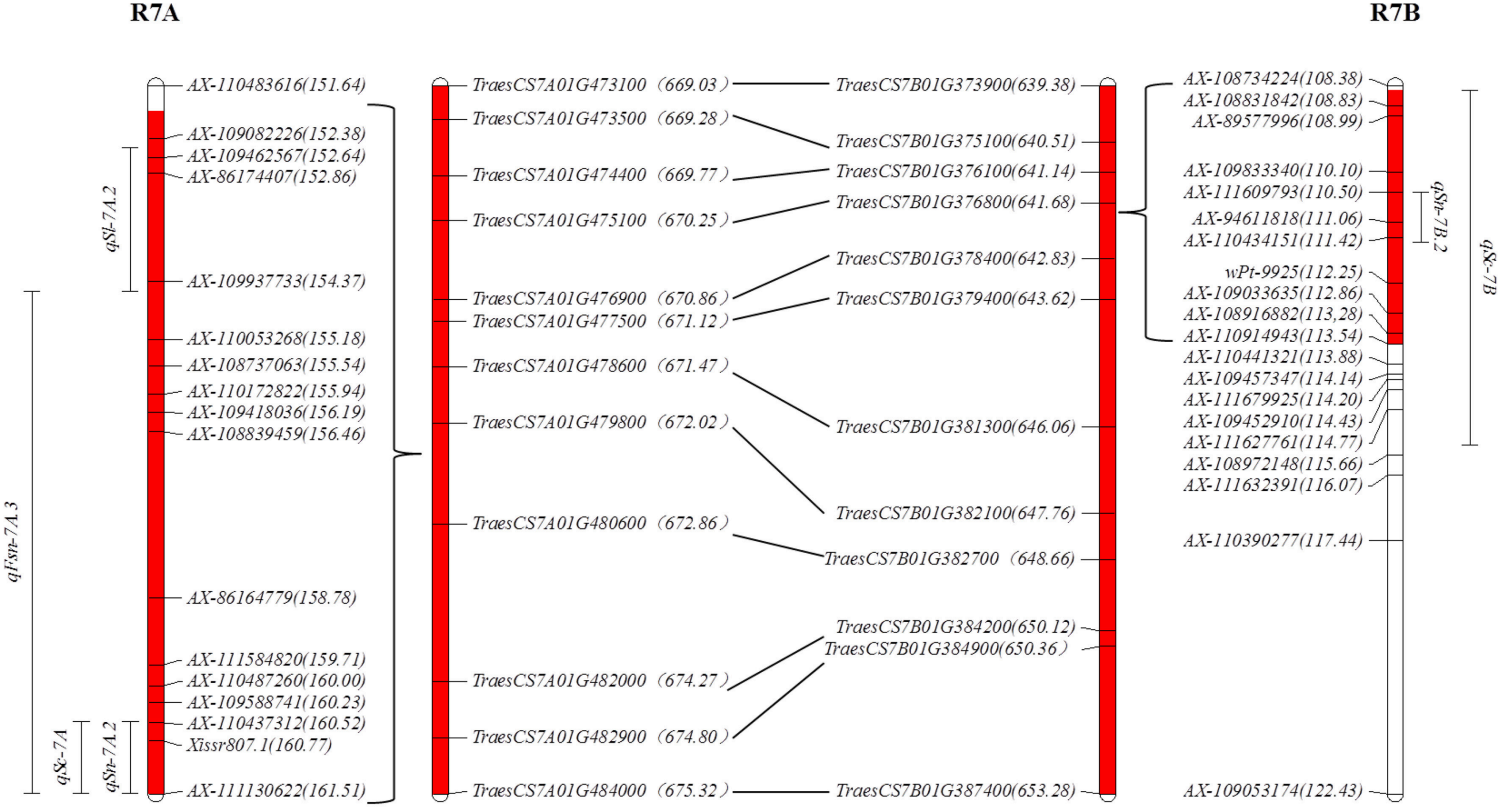
The syntenic region between R7A and R7B. The number in parentheses following the SNP marker (e.g., *AX-110483616*) indicates the genetic position (cm); the number in the parentheses following predicted gene (e.g., *TraesCS7A01G473100*) indicates the physical position (Mb); the red shadow indicates the range of the syntenic region between R7A and R7B.

*qSn-7A.2* and *qFsn-7A.3* in R7A (Table 3; Figure 1) were frequently reported to be associated with SN (Xu et al., 2014; Zhai et al., 2016; Giunta et al., 2018) and FSN (Liu et al., 2014; Zhai et al., 2016). However, the robust stable QTL controlling SC (*qSc-7A*) in R7A are presented for the first time in this study (Table 1). Spikelet compactness is a signature trait distinguishing club wheat from common wheat. The genes related to the clubbed head are useful in exploration of dissecting the relationship between spike morphology and sink capacity (Jantasuriyarat et al., 2004; Johnson et al., 2008). The discovery of stable QTL in R7A with strong and pleiotropic effect on SC, SN, and FSN might be valuable for facilitating spike morphology optimization in the breeding process. Because of the allopolyploid feature of common wheat, many major genes have homoeologues in the syntenic regions of the same homoeologous group (Khlestkina et al., 2008), such as dwarfing gene *Rht-B1* and *Rht-D1* localizing on 4BS and 4DS (Börner et al., 1997), respectively. In this paper, sixty-five genes in R7A were also found homoeologous genes in R7B whose CI nearby the orthologous to *CONSTANS* in barley (Table S9; Figures 1, 2), in consistence with the previous study (Giunta et al., 2018). This colinearity was possibly responsible for pairwise major homoeologous genes for spike characteristics on chromosomes 7A and 7B. The possibility that *CONSTANS* was the candidate gene involveing with R7B and R7A need further investigated.

Because no common markers or coincident QTL were found in R4A and R7D, these regions were regarded as having novel QTL controlling spike characteristics in this study (Table 3; Figure 1). In R4A, a major stable QTL for KN was previously detected by Cui et al. (2017) using the KJ-RIL population. J411 conferred the increased effect on HD, SN, FSN, and KN in R4A, indicating that the corresponding genes in this region might improve KN through modifying spike characteristics; thus, this pleiotropic region was important for high-yield breeding.

### Putative Pleiotropic Effect of *Rht-B1* on Spike Characteristics, Plant Type, and Kernel Formation

*Rht-B1* has commonly been reported to map to the QTL for yield parameters and grain quality (McCartney et al., 2005; Zhang et al., 2013b; Cui et al., 2016). However, except for Heidari et al. (2011), who located major QTL for spikelets/spike and SC close to *Rht-B1*, few reports specify its contribution to spike characteristics. Previously, using the same KJ-RIL population, an expected major stable QTL for PH (Zhang et al., 2017) and a major stable QTL for KN (Fan et al., 2015) were found to link with the *Rht-B1* locus. This study provides further preliminary confirmation of the putative pleiotropic effect of *Rht-B1* on spike characteristics (Table 4), which was consistent with the QTL mapping results (Table 3). This result revealed that *Rht-B1b* conferred decreased PH and SSN and contributed to the increased HD, SN, FSN, and KN, which were little affected by N application. This result indicated that *Rht-B1* might affect yield potential by controlling the plant type as well as sink capacity. However, *Rht-B1* was found to have a strong correlation with TKW but little association with KN when the Kauz/Westonia DH population by Zhang et al. (2013b). The inconsistency might have resulted from the use of different mapping populations and therefore deserves for further investigation.

## Conclusion

In this study, 157 and 54 spike-related loci were identified by traditional and conditional QTL mapping, respectively, based on ICIM. Among them, *qSn-1A.1, qFsn-1B.1*, and *qFsn-7D* were QTL completely induced by LN stress and their positive pyramiding effect on increasing KN was verified. Seven genomic regions were highlighted because they harbored stable QTL which could be detected by different detection models. Among them, R2D, R4B, R5A, and R7A harbored major stable QTL. R4A and R7D might contain novel QTL for spike characteristics. R7A and R7B show synteny in their candidate regions, implying that the homoeologous genes for spike characteristics possibly exist in R7A and R7B. Additionally, R2D, R4B, and R7B were found to be involved with *C, Rht-B1*, and wheat orthologous gene of *CONBSTAN*, respectively. *Rht-B1b* was validated to contribute to a significant reduction of PH and SSN but contributed to an increase of KN, HD, SN, and FSN, which explained the observed pleiotropy of R4B well. These LN-input sensitive loci and highlighted regions for spike-related traits could be helpful for improving the wheat sink capacity and yield potential.

## Author Contributions

XF, FC, and JL designed the research. XF and FC conducted genotyping of the KJ-RIL population. XF, FC, JJ, WZ, JL, DM, YT, TW, and JL conducted phenotyping of the KJ-RIL population. XF analyzed data and wrote the paper. TW and JL had primary responsibility for final content. All authors read and approved the final manuscript.

### Conflict of Interest Statement

The authors declare that the research was conducted in the absence of any commercial or financial relationships that could be construed as a potential conflict of interest.

## Abbreviations

SN: spikelet number
FSN: fertile spikelet number
SSN: sterile spikelet number
SC: spike compactness
SL: spike length
HD: heading date
KN: kernel number per spike
PH: plant height
KJ-RILs: recombinant inbred line population derived from the cross between Kenong9204 and Jing411
N: nitrogen
LN: low nitrogen
HN: high nitrogen
QTL: quantitative trait loci.
